# Secure Smart Wearable Computing through Artificial Intelligence-Enabled Internet of Things and Cyber-Physical Systems for Health Monitoring

**DOI:** 10.3390/s22031076

**Published:** 2022-01-29

**Authors:** Lakshmana Kumar Ramasamy, Firoz Khan, Mohammad Shah, Balusupati Veera Venkata Siva Prasad, Celestine Iwendi, Cresantus Biamba

**Affiliations:** 1 Hindusthan College of Engineering and Technology, Coimbatore 641032, India; research.laksha@gmail.com; 2 Higher Colleges of Technology, Dubai 15825, United Arab Emirates; fk7@hotmail.com; 3 School of Creative Technologies, University of Bolton, Bolton BL3 5AB, UK; m.shah@bolton.ac.uk (M.S.); celestine.iwendi@ieee.org (C.I.); 4 School of Engineering, Mallareddy University, Hyderabad 500100, India; drbvvsivaprasad@gmail.com; 5 Department of Educational Sciences, Faculty of Education and Business Studies, University of Gavle, 801 76 Gävle, Sweden

**Keywords:** Internet of Computers, Internet of Things, Cyber-Physical System, artificial intelligence, patients, classification

## Abstract

The functionality of the Internet is continually changing from the Internet of Computers (IoC) to the “Internet of Things (IoT)”. Most connected systems, called Cyber-Physical Systems (CPS), are formed from the integration of numerous features such as humans and the physical environment, smart objects, and embedded devices and infrastructure. There are a few critical problems, such as security risks and ethical issues that could affect the IoT and CPS. When every piece of data and device is connected and obtainable on the network, hackers can obtain it and utilise it for different scams. In medical healthcare IoT-CPS, everyday medical and physical data of a patient may be gathered through wearable sensors. This paper proposes an AI-enabled IoT-CPS which doctors can utilise to discover diseases in patients based on AI. AI was created to find a few disorders such as Diabetes, Heart disease and Gait disturbances. Each disease has various symptoms among patients or elderly. Dataset is retrieved from the Kaggle repository to execute AI-enabled IoT-CPS technology. For the classification, AI-enabled IoT-CPS Algorithm is used to discover diseases. The experimental results demonstrate that compared with existing algorithms, the proposed AI-enabled IoT-CPS algorithm detects patient diseases and fall events in elderly more efficiently in terms of Accuracy, Precision, Recall and F-measure.

## 1. Introduction

Artificial Intelligence (AI) has grown to be very popular in today’s world. AI aims to transform computers into rational human beings. This growth would speed up the adoption of digital technology to services or businesses of industries. Combining humans, machinery, plants, animals, equipment, soil, rocks, ponds, constructions or whatever one can think of, and taking “intelligent decisions” would make the world an independent place. For the world and its physical body to become truly independent, we require machine learning (ML) [1] to follow a process of acquiring knowledge and a set of data analysis (DA) [2].

ML would develop abilities for automating and self-levelling learning across different devices on the network. Simultaneously, DA would assess the entire data, create more time to detect former trends and is very efficient in creating upcoming trends. This trend is rising, and attempts to integrate DA and ML into sensors [3] and embedded systems [4]. AI technology is genuinely fascinating, and it inspires us to consider again all the things we recognize regarding the intention of work and life. The speed at which ML and DA run AI, invites for a well-structured framework to debate emerging trends, threats and challenges.

One of the great concepts behind this trend is the Internet of Things (IoT) [5], which consists of a world full of sophisticated gadgets to install, often referred to as “smart objects” [6] that overlap with other media such as an Internet, Bluetooth and so on. These links are physical-physical things, human-physical things, and human-human. The Internet of Everything [7] is the same concept, meaning that each living, inanimate or virtual object is linked to everyone and has some means of contact.

When these concepts apply to the world of physics, we get the Cyber-Physical System (CPS) [8]. Such a world may have rich data from which information can be extracted. Different fields such as Machine Learning (ML), Database Management System (DBMS) [9], Data Mining (DM), Pattern Recognition (PR) [10], and Big Data Analytics (BD) [11] require advanced techniques of dealing with data, which vary widely in their range. Recent years have seen China enter the aging community. The problem of population aging is becoming more and more acute. Physical conditions of the elderly are declining, including the ability to maintain heart function and gait balance. Health care and safety monitoring for the elderly is becoming an urgent issue that needs to be addressed. The existing smart wearable computing and remote patient monitoring systems can monitor and detect patient disease and fall events in elderly efficiently for small dataset. To deal with large amount of data, this paper focuses on AI-enabled IoT-CPS that doctors can utilize to discover diseases in patients or elderly persons with security. In AI-enabled IoT-CPS, both ML and Deep Learning (DL) algorithms are used for AI.

The remaining section of the paper is organized as follows: The related works about IoT, CPS and AI are reviewed in Section 2 and also it describes the process of AI-enabled IoT-CPS for smart wearable computing. The results of the experiments are debated in Section 3. Finally, Section 4 concludes the paper.

## 2. Materials and Methods

This section discusses the related works about AI in IoT and AI in CPS.

Currently, the ideas of robots and AI are extensively familiar, and AI has excellent benefits for human lives and research. Accompanied by the advancement of the application of science, especially to industrial objectives, AI has transformed into a notable growth trend. Simultaneously, the Internet is gradually spreading, driving the fast growth of all stages of life. In this context, the IoT was coined, and its dynamic role in the growth of China’s economy continues to fascinate us. Ref. [12] Conducted extensive research into the IoT using a variety of applications of AI, such as robots, computer networks, smart homes, and self-driving cars, and its application potential was debated.

Ref. [13] proposed a growth framework for smart animal management systems using IoT and AI. Its primary purpose is to automate a few complicated processes for animal-care by IoT and AI, protecting animal managers and allowing them to control the animals very formally.

Ref. [14] suggested Wireless Home Automation System (WHAS) based on IoT that uses mobile devices and a microprocessor to spontaneously handle fundamental functions and characteristics of a house online from any place globally. The home automation was named the “Smart Home”. It was designed to minimize environmental impact. The home automation application differs from other applications by permitting the consumer to use the application via the Internet. Ref. [15] Authors discussed Blockchain-based Confidentiality-Privacy preserving Big Data scheme for healthcare clouds and applications. In [16] the concept of ambient acoustic event assistive framework for identification, detection, and recognition of unknown acoustic events of a residence was studied.

Ref. [17] described a novel process for executing AI, Cloud and IoT in robots. Presently, AI plays a significant role in the robotics world. Nearly every industry uses robots to perform a variety of tasks. However, there have been a few issues with the creation of a multi-task robot. Thus, there is a necessity to develop a novel way of creating multi-task robots, which can only be done with AI and IoT. Being linked to the cloud will minimize cost. The author explains the procedure of making a robot.

When AI drives things on the Internet, it is referred to as the Internet of Intelligent Things. Nathan et al. [18] debated on the fundamental parts of the Internet of Intelligent Things and a few of the significant areas of application where it is utilized. Authors presented a complete overview of the IoT for start-ups, business managers and policymakers for the future. Recognizing and prediction of the spread of contagion based on mobile phone location data was analyzed using artificial intelligence concepts [19].

Ref. [18] proposed an Artificial Intelligence of Things (AIoT) technique for the ECG research and heart disease diagnosis. The method contains a user interface hardware based on IoT, a front-end on the Smart Device Application (APP), a cloud database, and an AI platform for cardiac diagnostics. A user interface hardware based on IoT, portable ECG connector, contains an analogue user interface circuit and Bluetooth module that could discover ECG signals. APP on smart devices could not solely show consumer’s actual-time ECG signals; however, they can quickly label abnormal movements and achieve actual-time diagnoses. These ECG signals are transmitted to the database of the cloud. The database of the cloud is utilized to save individual consumers ECG signals, creating a database of big data for the algorithm of AI to discover heart diseases. The algorithm presented by the authors uses a Convolution Neural Network, and the mean precision is 94.96%. The dataset of ECG used for this algorithm was gathered from patients at Tainan Hospital. Also, signal confirmation was further executed by heart specialists.

AI techniques, such as deep learning techniques are progressively being utilized to optimize the functioning of parts of CPS. Accompanied by the growth of the domain of AI, CPS is evolving because the work excellence of similar systems is mostly decided by the greatness of processing data in the system. Plakhotnikov et al. [20] utilized the AI in CPS. 

The authors in [21] analyzed various Cyber physical systems in the smart city perspective. Deep learning model construction for a semi-supervised classification with feature learning was done in [22]. The detailed healthcare application was developed by [23] which use Blockchain and Business Process Management in Health Care, Especially for COVID-19 Cases. The idea of using Hybrid Reality-Based Education Expansion System for Non-Traditional Learning in [24]. Trust-Aware Routing Framework for Internet of Things was discussed in [25].

Ref. [26] presented a hybrid intelligent-classic control technique to reconstruct and compensate for cyber-attacks initiated by non-linear CPS and industrial IoT operating via resource-sharing computer communications networks. In this scenario, CPS is considered a type of n-order non-linear application when cyberattacks are present solely in the forward channel. In this plan, the Gaussian radial basis functions NN utilized for online evaluation and reconstruction of cyber-attacks started on a networked computer. The law of adaptation of the wise evaluator was obtained from the work of Lyapunov.

Ref. [27] demonstrated the importance of growing a CPS for technical condition detection technique for apparatuses Generation of high voltages. The very susceptible component of a similar machine is winding-insulation. The simplest and cheapest technique to determine this error is the research of the Electro-Discharge Activity (EDA). This technique allows the determination of the insulation defect at an early phase of its growth. One of the significant issues in creating apparatus detection is the substantial distance of its components from each other. A cyber-physical prediction diagnostic technique has been proposed to resolve this issue. A diagnostic approach for creating apparatus windings was also suggested. This technique allows the determination of the generator insulation’s technical condition throughout its function in the absence of complex computing devices based on AI.

Furthermore, ref. [28] proposed SALP swarm algorithm based automatic image classification technique. In addition [29,30] discussed liver segmentation using deep learning networks and gated power optimization for biomedical therapeutic devices respectively. Furthermore, ref. [31] discussed on nutrition recommendation using optimization techniques for diabetic patients. 

In addition, some works focused on security especially [32,33] proposed a framework Cyber-security risk assessment in critical infrastructures and mass scan networks. Additionally, ref. [34] proposed a blockchain based secure storage mechanism for WSN. In addition, ref. [35] discussed some security plans against network attacks in CPS. Enhancing security of health information using modular encryption standard in mobile cloud computing was discussed in [36]. Artificial intelligence enabled road vehicle-train collision risk assessment framework for unmanned railway level crossings proposed by [37]. Authors in [38] discussed about realizing the efficient security and privacy in IoT networks.

### 2.1. Secure Smart Wearable Computing

Large or small-scale data is a necessary segment of the IoT world of linked devices. Smart objects can do a small amount of local processing and need to have several innate intelligences. In whatever way, for a data-based conclusion, a lot of data must be used. It is not always probable to store this data for analysis within the smart object. In this situation, a macroscopic scale comes into operation; information is transmitted to distant places in a shared format and examined. Results of the research are then consolidated, and a final product can, in some cases, be returned to a smart object where an actuator could execute its function. The time between transmitting data and processing a decision must be realistically short; otherwise, it will not make sense. Conventional data analysis tools are not efficient in catching the full substance of this extensive data in actual time. Also, the speed, volume, and type are excessively massive for detailed research. In contrast, the scale of possible connections and interactions between various data sources is too wide for any researcher to understand manually. AI system is needed to deal with such extensive data.

1. Data processing skills
2. Learning Methods—Advanced and Basic
3. Adaptation and automation procedures
4. Measurability
5. Group modelling
6. Actual-time decision-making.

It means that the system can construct most of the choices and take the necessary steps rapidly. ML already has an excellent ability to allow you to do a few thinking about computers. However, when we try to deal with big data, we try to get more and more data. That is why we require AI techniques to manage big data and come up with a few novel plans. 

### 2.2. AI-Enabled IoT-CPS

The origins of CPS and IoT are encouraged by the plans of economic and social benefits. Thus, CPS and IoT could be applied as smart transport, smart businesses, smart grid and personalized healthcare and so on. For instance, the intelligent industry could enhance its production procedures by distributing actual-time data between different industrial tools, distributors, supply chains, business organizations and consumers. A healthcare CPS such as a smart hospital can remotely monitor patients’ physical conditions. On a road accident, the family member, police station and nearest hospital can be alerted. An ambulance would be instantly transported to the spot of the accident, with the doctor on duty notified, and without wasting any time, the police may reach the area to do things manually. Some emergencies would benefit more from these connected independent systems. It is this ‘intelligence’ that AI brings with it, such as in this IoT-CPS infrastructure.

IoT-CPS applications include elements that communicate with complicated physical surroundings. Such an integrated surrounding is a demanding discovery that could change existing efforts. For instance, agriculture, security, emergency response systems, vital infrastructure, buildings, transportation, health care, energy systems, manufacturing industries, and so on would upgrade to their best and connected versions.

Such companies must have system awareness assets that will spontaneously confirm future bugs or failure on the system. Being a system alert means that a device fitted to any equipment segment can sense itself with its surroundings. Improvements in AI for the linked IoT-CPS framework assist in recognizing and enhancing the vision of the “Intelligent Planet”.

Even though machines were not designed to replace humans completely, they do assist humans in decreasing their workload. Humans require maintaining dominance over machines. AI is more efficient when combined with human intelligence. It highlights those humans and computers have various strengths in the broader field of excellence; computers are more effective at math tasks and numbers, while humans show significant performance in logic and reasoning. These different formats of excellence are perfect. So, AI is a technology that could achieve our dream of having “thinkable” things.

**Figure 1:**
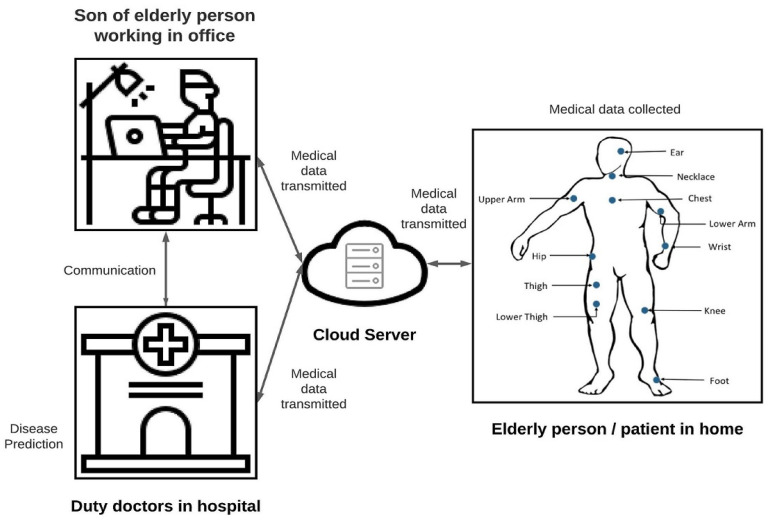
AI-enabled IoT-CPS.

Figure 1 illustrates medical healthcare IoT-CPS application comprised of three main sites, namely: the home, hospital and office environment. The purpose of this developed application is to improve the Quality of Life (QoL) of an older person (or patients) whose son is working in the office at daytime and to save medical cost. In a normal situation, the daily physical and medical information of an older adult is collected (by the camera, biosensor etc.) and stored in a third-party cloud (like the central lab) via an in-home WSN-Cloud Gateway. The son in the office and hospital doctors (or nurses) can regularly check such medical record and apply AI to detect diseases, and give some suggestions and prescriptions via a wired or wireless connection to the cloud in an authenticated way. In an urgent situation (like if the older adult falls), such emergent information will be sent promptly to both the doctors and their family members so that immediate actions can take to help the fallen elder.

Furthermore, security and trust in linked devices are two critical issues for the greater acceptance of IoT-CPS. IoT-CPS is very emphatic on being a safe choice for users to distribute their data. When every piece of data and device is connected and obtainable on the network, it could be obtained by hackers and utilized for different scams. For instance, an IoT-CPS linked house may raise the danger of a burglary. Or the privacy of a patient may be infringed when opponents obtain their disease data.

To improve security, the son of the older adult may generate a public key and private key. He/she shares his/her public access key to each wearable device and shares his/her private key with the hospital duty doctor. After sensing, these devices encrypt sensed data based on public key and shares encrypted data to the son and doctor. After getting cipher text, son and doctor can decrypt and access data using the private key.

**Figure 2:**
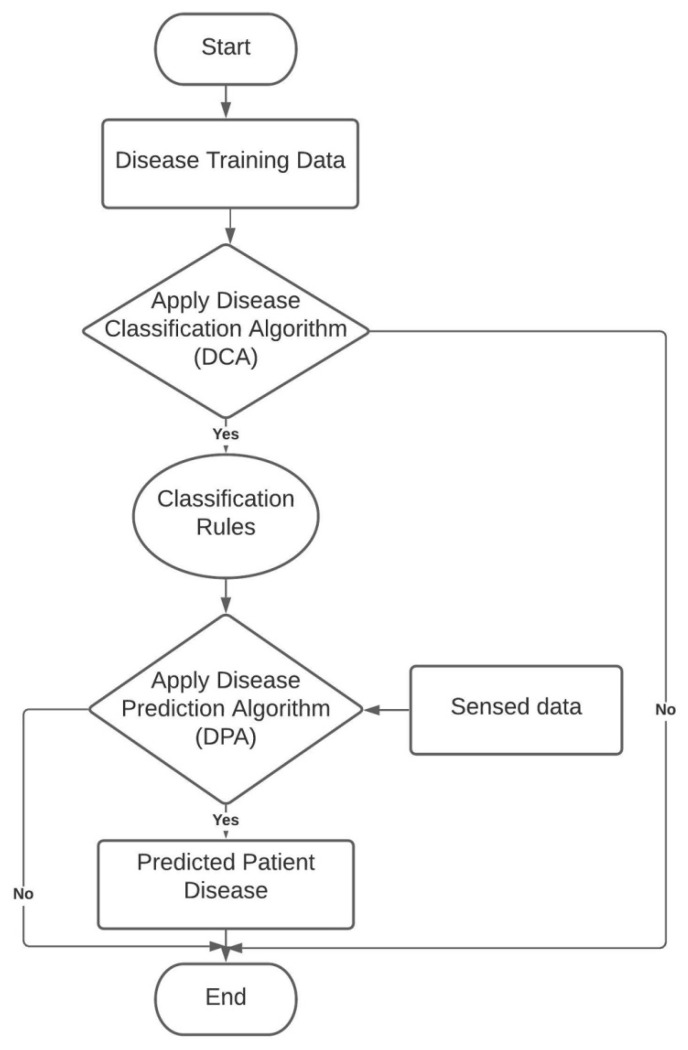
Flow chart of AI-enabled IoT-CPS Algorithm.

In our society, physicians are responsible for diagnosing, treating or controlling the disease and giving medical treatments or therapy. A few disorders may be cured through treatment; however, chronic diseases may not ever be cured. Treatment can however avert the chronic diseases from getting worse in the long run. Therefore, it is very important to diagnose and treat the disease at an early stage. In medical healthcare IoT-CPS, everyday medical and physical data of a patient or older adults are gathered through wearable sensors. This chapter proposed AI-enabled IoT-CPS that doctors can utilize to discover diseases in patients based on AI with security. AI was created to find a few disorders, such as Chronic Kidney disease, Diabetes, Heart disease, Hepatitis and Liver disorders. Each disease has various symptoms among patients. Multiple datasets are retrieved from the UCI machine learning repository to execute AI-enabled IoT-CPS technology. For the classification, AI-enabled IoT-CPS Algorithm is used to discover diseases. This algorithm is categorized into two sub-algorithms. The first sub-algorithm is Disease Classification Algorithm (DCA) which classifies the patient’s disease training dataset and generates some classification rules. The second sub-algorithm is Disease Prediction Algorithm (DPA) which predicts the patient’s diseases for disease testing dataset based on classification rules. Figure 2 illustrates the flowchart of the AI-enabled IoT-CPS Algorithm.

#### 2.2.1. Disease Classification Algorithm

This algorithm classifies patients’ diseases from training dataset and generates some classification rules. Algorithm 1 shows the DCA workflow. This algorithm first extracts all instances from patient’s disease training dataset (Step 1). A single row of data is called an instance. Next, it obtains the target attribute (class) (Step 2). Furthermore, it extracts all the target attribute values from each instance (Step 3). Then, it gets other attributes from each row (Step 4). This algorithm first calculates the global disorder amount from all target attribute values (Steps 5–10).
**Algorithm 1: DCA: Disease Classification Algorithm****Input****:**Disease Training Data (DTrD)**Output****:**Classification Rules (ClassiRule1, ClassiRule2, ClassiRule3)**Step 1****:**R[] <-- Extract all instances from DTrD**Step 2****:**TA <-- Extract target attribute from R**Step 3****:**TAV[] <-- Extract target attribute values from R**Step 4****:**OA[] <-- Extract other attributes from R**Step 5****:**GDA = 0              //Global Disorder Amount**Step 6****:**For each TAVi from TAV**Step 7****:**   F <-- Count Frequency of TAVi**Step 8****:**   FP <-- F/R.length        //Frequency Probability**Step 9****:**   GDA <-- GDA − (FP * log2(FP))**Step 10****:**End For**Step 11****:**ClassiRule1 = ClassificationRuleGeneration(OA,R)**Step 12****:**ClassiRule2 = Classify DTrD based on MLP classifier using weka**Step 13****:**ClassiRule3 = Classify DTrD based on Dl4jMlpClassifier using WekaDeeplearning4j**Step 14****:**Return ClassiRule1, ClassiRule2, ClassiRule3

This algorithm applies the Classification Rule Generation Method (Step 11) to generate classification rules (ClassiRule1) based on GDA, OA and R. Furthermore, this algorithm used MLP classifier and Dl4jMlpClassifier (for AI) for Disease Training Data classification. These both classifiers provide ClassiRule2 and ClassiRule3 respectively.
**ClassificationRuleGeneration(OA,R)****Input****:**OA, R**Output****:**ClassiRule1**Step 1****:**TAV[] <-- Extract target attribute values from R**Step 2****:**IF (OA.length = 0)**Step 3****:**   i = 0, highestCount = 0, highestTAVi = ““**Step 4****:**   For each TAVi from TAV**Step 5****:**      F <-- Count Frequency of TAVi**Step 6****:**      IF i = 0**Step 7****:**         highestCount = F**Step 8****:**         highestTAVi = TAVi**Step 9****:**      ELSE IF (highestCount < F)**Step 10****:**         highestCount = F**Step 11****:**         highestTAVi = TAVi**Step 12****:**      End IF**Step 13****:**   End For **Step 14****:**   ClassiRule1.Attribute = TA**Step 15****:**   ClassiRule1.classValue = highestTAVi//Highest Target Attribute Value**Step 16****:**   Return ClassiRule1**Step 17****:**ELSE IF (TAV.length = 1)**Step 18****:**   ClassiRule1.Attribute = TA**Step 19****:**   ClassiRule1.classValue = TAV [0] //Target Attribute Value**Step 20****:**   Return ClassiRule1**Step 21****:**End IF**Step 22****:**AODAS = {}**Step 23****:**count = 0**Step 24****:**For each Attribute OAi from OA**Step 25****:**   OAiV[] <-- Extract OAi values from CDTrD**Step 26****:**   LDA = 0            //Local Disorder Amount**Step 27****:**   For each Value OAiVj from OAiV**Step 28****:**      F <-- Count Frequency of OAiVj**Step 29****:**      FP <-- F/R.length     //Frequency Probability**Step 30****:**      LDA <-- LDA − (FP * log2(FP))**Step 31****:**   End For**Step 32****:**   OAiDAScore = GDA − LDA**Step 33****:**   AODAS[count] = OAiDAScore**Step 34****:**   count ++**Step 35****:**End For**Step 36****:**Sort AODAS based on ascending order**Step 37****:**HPA <-- AODAS[0]  //Highest Priority Attribute**Step 38****:**//Because which attribute has minimum OAiDAScore, that has the highest priority**Step 39****:**ClassiRule1.Attribute = HPA**Step 40****:**newOA[] = new int[OA.length − 1]**Step 41****:**pos = 0**Step 42****:**For each Attribute OAi from OA**Step 43****:**   If (OAi ! = HPA)**Step 44****:**      newOA[pos ++] = OAi**Step 45****:**   End If**Step 46****:**End For**Step 47****:**//Partition the Data Set Based on the Values of the HPA Attribute**Step 48****:**OAiV[] <-- Extract HPA values from CDTrD**Step 49****:**PARTI[] = {}, pcou = 0**Step 50****:**For each value VAL from OAiV**Step 51****:**   listInstances[] = {}, cou = 0**Step 52****:**   For each instance INS from R**Step 53****:**      If (INS contains VAL)**Step 54****:**         listInstances [cou] = INS**Step 55****:**         cou ++**Step 56****:**      End If**Step 57****:**   End For**Step 58****:**   PARTI [pcou] = VAL,listInstances**Step 59****:**   pcou ++**Step 60****:**End For**Step 61****:**ClassiRule1.attributeValues = String [PARTI.size()]**Step 62****:**ClassiRule1.attributes = Attribute [PARTI.size()]**Step 63****:**index = 0**Step 64****:**For each partition PAR from PARTI**Step 65****:**   VAL = PAR.getKey()**Step 66****:**   newR = PAR.getValue()   //listInstances**Step 67****:**   ClassiRule1.attributeValues [index] = VAL**Step 68****:**   ClassiRule1.attributes [index] = ClassificationRuleGeneration(newOA, newR)//recursive call**Step 69****:**   index + +**Step 70****:**End For**Step 71****:**Return ClassiRule1

It takes other attributes such as the global disorder amount, target attribute and target attribute values as input. This algorithm first finds The Highest Priority Attribute (HPA) among all features based on disorder amount score (Steps 22–37). HPA refers to what column has a minimum disorder amount score that has the highest priority. Next, this algorithm partitions the dataset based on HPA attribute (Steps 39–60). Furthermore, this algorithm applies the Classification Rule Generation Method for each partition repeatedly (Steps 40–70) until all attributes taken as HPA attribute. There is no HPA attribute; this algorithm finds the highest target attribute value. After finding the highest target attribute value, this algorithm returns all HPA attributes and its values with target attribute value as classification rules (Steps 1–21).

#### 2.2.2. Disease Prediction Algorithm

This algorithm predicts patients’ diseases based on classification rules. Algorithm 2 explains the DPA Algorithm. Furthermore, this algorithm takes Patient Disease Testing Data from patients or elderly persons’ wearable devices and classification rules as input. This algorithm extracts all attribute values from the received data. It compares attribute values with classification rules and determines what decision it should take (Steps 3–15). Furthermore, MLP classifier algorithm predicts patient disease for testing dataset using ClassiRule2 and Dl4jMlpClassifier algorithm predicts patient disease for testing dataset using ClassiRule3. Among these 3 predictions, the majority is winning.
**Algorithm 2: Disease Prediction Algorithm (DPA)****Input****:**Testing data, Classification Rules (ClassiRule1, ClassiRule2 and ClassiRule3)**Output****:**Predicted Patient Disease (PRED)**Step 1****:**//If this attribute is a target attribute, then return the target attribute value**Step 2****:**IF(ClassiRule1.attribute = TA)**Step 3****:**   PRED1 = ClassiRule1.classValue**Step 4****:**ELSE**Step 5****:**   //otherwise, check which rule should follow**Step 6****:**   //by comparing the attribute of the instance**Step 7****:**   //with the one in the rule**Step 8****:**   VAL = INS[ClassiRule1.attribute]**Step 9****:**   i = 0**Step 10****:**   For each ClassiRule1.attributeValues [i] from ClassiRule1.attributeValues**Step 11****:**      IF (ClassiRule1.attributeValues [i] == VAL)**Step 12****:**         predictTargetAttributeValue(ClassiRule1.attributes [i], INS)**Step 13****:**      End IF**Step 14****:**   End For**Step 15****:**End IF**Step 16****:**PRED2 = Predict Testing data using MLP classifier with ClassiRule2**Step 17****:**PRED3 = Predict Testing data using Dl4jMlpClassifier with ClassiRule3**Step 18****:**PRED = ““**Step 19****:**IF (PRED1 = PRED2)**Step 20****:**   PRED = PRED1**Step 21****:**ELSE IF (PRED1 = PRED3)**Step 22****:**   PRED = PRED1**Step 23****:**ELSE IF (PRED2 = PRED3)**Step 24****:**   PRED = PRED2**Step 25****:**End IF**Step 26****:**return PRED

## 3. Results

Some of the necessary sensors are attached to the elderly person’s body and takes readings including motion, temperature, and so on to verify the proposed effectiveness of the AI-enabled IoT-CPS algorithm. The doctor analyzes these data, detects the disease or falls manually and stores it in its database. This database is called the training dataset. In this study, Remote monitoring disease dataset is generated based on [39]. After he generates the training dataset, he can detect disease or fall event automatically and quickly based on the AI-enabled IoT-CPS algorithm.

The proposed AI-enabled IoT-CPS algorithm also evaluated a fall detection dataset. The fall detection dataset obtained from the Kaggle data repository contains elderly patients’ activity and medical information [40]. As a result, four different fall trajectories (right, left, backwards and forward), three standard activities (lying down, walking and standing) and situations close to the fall were discovered. Falls are a severe public health problem and could be life-threatening for those in the fall risk groups. This section implements an automated fall discovery system with wearable motion sensor units mounted on the subject’s body at six different stages. Each unit consists of three tri-axial devices (compass/magnetometer gyroscope and accelerometer). Fourteen volunteers do standardized movements, including 20 voluntary falls and 16 daily life activities (ADL), resulting in a huge dataset with 2520 trials.

Next, four existing disease prediction algorithms, namely Naïve Bayes, SVM, KNN, and ANN [41] are compared with the proposed AI-enabled IoT-CPS algorithm in terms of Accuracy, Precision, Recall, F-measure and Execution time. Table 1 shows the accuracy results of different algorithms for disease prediction.

**Figure 3:**
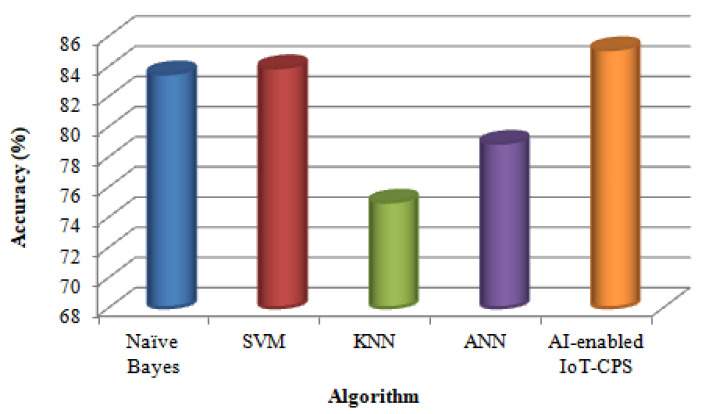
Comparison based on accuracy.

Figure 3 shows accuracy comparisons of different algorithms for disease prediction.

Figure 3 shows that compared with the KNN algorithm, the ANN algorithm accuracy is high. This is because in an extensive training dataset, the KNN algorithm takes more time to find the nearest neighbors. But compared with ANN, Naïve Bayes algorithm provides the highest accuracy. If the neural network is extensive, the ANN algorithm requires high processing time. Furthermore, compared with Naïve Bayes, SVM algorithm accuracy is high. Because the amount of data is less, the accuracy of the Naïve Bayes algorithm decreases. But compared with SVM, the proposed AI-enabled IoT-CPS algorithm provides the highest accuracy because speed and size requirement both in training and testing is more in the SVM algorithm.

This work also compares the four algorithms with the proposed AI-enabled IoT-CPS algorithm in terms of Precision. Table 2 shows various classification algorithms in precision results.

**Figure 4:**
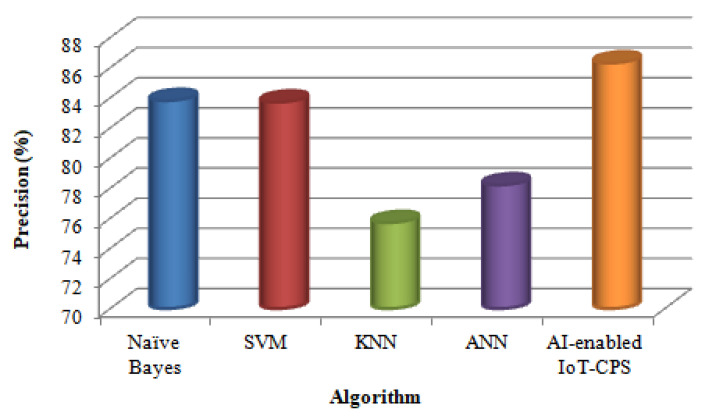
Comparison based on precision.

Figure 4 shows the results of the experiment for the four algorithms with the proposed AI-enabled IoT-CPS algorithm.

Figure 4 shows that compared with the KNN algorithm, the ANN algorithm precision is high. The KNN algorithm is sensitive to noisy or irrelevant attributes. But compared with ANN, SVM algorithm provides the highest precision. This is because the ANN algorithm was challenging to know the amount of neurons and layers necessary. Furthermore, compared with SVM, Naïve Bayes algorithm precision is high. This is because of high complexity and extensive memory requirements for classification in many cases in the SVM algorithm. But compared with Naïve Bayes, the proposed AI-enabled IoT-CPS algorithm provides the highest precision. This is because the Naïve Bayes algorithm requires a vast number of records for it to obtain good results.

Furthermore, this work compares the four algorithms with the proposed AI-enabled IoT-CPS algorithm in terms of Recall. Table 3 shows various classification algorithms recall results.

**Figure 5:**
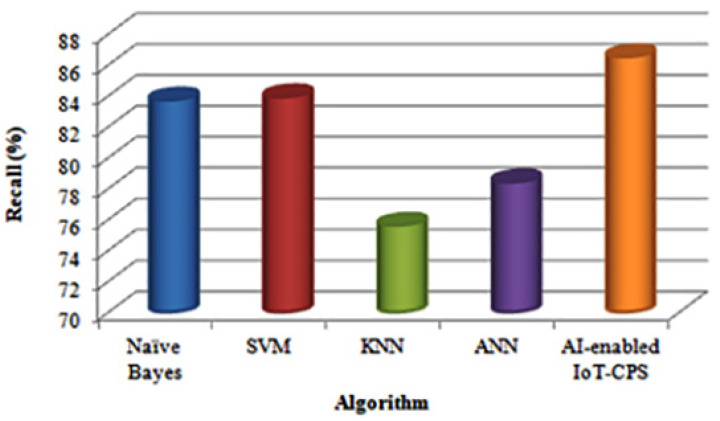
Comparison based on recall.

Figure 5 shows the recall when using the Naïve Bayes, SVM, KNN, ANN [13] and the proposed AI-enabled IoT-CPS algorithm.

Figure 5 shows that compared with the KNN algorithm, ANN’s recall is high. KNN algorithm performance depends on the number of dimensions used. But compared with ANN, Naïve Bayes algorithm provides the highest recall. ANN algorithm Learning is too slow. Furthermore, compared with Naïve Bayes, SVM algorithm recall is high. This is because the amount of data is less, so the recall of the Naïve Bayes algorithm decreases. But compared with SVM, the proposed AI-enabled IoT-CPS algorithm provides the highest recall. This is because, when the dataset is too large, SVM doesn’t perform very well.

Finally, this work compares the four algorithms with the proposed AI-enabled IoT-CPS algorithm in terms of F-Measure. Table 4 shows various classification algorithms F-Measure results.

**Figure 6:**
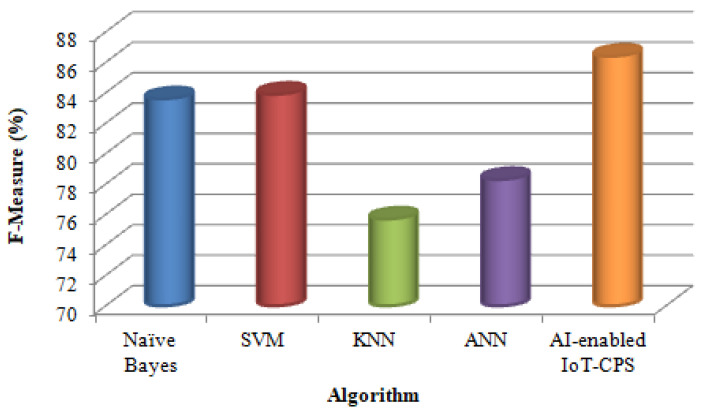
Comparison based on F-Measure.

Figure 6 shows the F-measure value when using the Naïve Bayes, SVM, KNN, ANN [13] and the proposed AI-enabled IoT-CPS algorithm.

Figure 6 shows that compared with the KNN algorithm; ANN’s F-measure is high. This is because finding the optimal “k” value is difficult in the KNN algorithm. But compared with ANN, Naïve Bayes algorithm provides the highest F-measure, for the reason that ANN algorithm takes a long time for substantial neural network processing. Furthermore, compared with Naïve Bayes, SVM algorithm F-measure is high. This is because Naïve Bayes algorithm requires the removal of irrelevant features for obtaining a high F-measure value. But compared with SVM, the proposed AI-enabled IoT-CPS algorithm provides the highest F-measure because SVM takes more time to process classification and prediction.

## 4. Conclusions

This paper proposed an AI-enabled IoT-CPS that doctors can utilize to detect diseases in patients based on AI with security. Machines are not designed to replace humans completely, however, they do assist humans in decreasing their workload. Humans need to maintain dominance over machines. AI is more efficient when combined with human intelligence. It highlights that humans and computers have various strengths in the broader field of excellence; computers are more effective at math tasks and numbers, while humans show significant performance in logic and reasoning. These different formats of excellence are perfect. So, AI could achieve our dream of having “thinkable” things. The proposed AI-enabled IoT-CPS algorithm are categorized into two sub-algorithms. The first sub-algorithm is Disease Classification Algorithm (DCA) which classifies the patients’ disease training dataset and generates some classification rules. The second sub-algorithm is Disease Prediction Algorithm (DPA) which predicts the patients’ diseases for disease testing dataset based on classification rules. The experimental results showed the proposed AI-enabled IoT-CPS algorithm is more effective for diagnosing the patient’s disease than existing algorithms in terms of Accuracy, Precision, Recall, F-measure and Execution time. Data protection was not considered in this proposed AI-enabled IoT-CPS algorithm. The huge quantity of information distributed over various IoT and CPS devices makes these devices a target for fraudsters, attackers, and various unethical users fascinated with such information. If this information falls into the wrong hands, it could cause great harm to those involved. Therefore, security considered AI-enabled IoT-CPS algorithm is needed for the future.

## Figures and Tables

**Table 1:** Accuracy values of different classification algorithms.

Algorithm	Accuracy (%)
Naïve Bayes	83.5
SVM	83.9
KNN	75.0
ANN	78.9
AI-enabled IoT-CPS	85.1

**Table 2:** Precision values of different classification algorithms.

Algorithm	Precision (%)
Naïve Bayes	83.8
SVM	83.7
KNN	75.7
ANN	78.2
AI-enabled IoT-CPS	86.3

**Table 3:** Recall values of Different Classification Algorithms.

Algorithm	Recall (%)
Naïve Bayes	83.7
SVM	83.9
KNN	75.6
ANN	78.4
AI-enabled IoT-CPS	86.5

**Table 4:** F-Measure values of Different Classification Algorithms.

Algorithm	F-Measure (%)
Naïve Bayes	83.6
SVM	83.9
KNN	75.7
ANN	78.3
AI-enabled IoT-CPS	86.4

## Data Availability

The data that support the findings of this study are unavailable in any public repositories.
